# Positive personal resources and psychological distress during the COVID-19 pandemic: resilience, optimism, hope, courage, trait mindfulness, and self-efficacy in breast cancer patients and survivors

**DOI:** 10.1007/s00520-022-07123-1

**Published:** 2022-05-17

**Authors:** Francesca Chiesi, Deborah Vizza, Moira Valente, Rosy Bruno, Chloe Lau, Maria Rosita Campagna, Melania Lo Iacono, Francesco Bruno

**Affiliations:** 1 Department of Neuroscience, Psychology, Drug, and Child’s Health (NEUROFARBA), Section of Psychology, University of Florence, Via S.Salvi 12, 50135 Florence, Italy; 2 Academy of Cognitive Behavioral Sciences of Calabria (ASCoC), Lamezia Terme, Italy; 3 Voluntary Association “Ali Rosa”, Rende, CS Italy; 4 “Annunziata” Hospital, Cosenza, Italy; 5 Department of Psychology, Western University, London, ON Canada; 6 Regional Neurogenetic Centre (CRN), Department of Primary Care, Azienda Sanitaria Provinciale Di Catanzaro, Viale A. Perugini, 88046 Lamezia Terme (CZ), Italy; 7 Association for Neurogenetic Research (ARN), Lamezia Terme (CZ), Italy

**Keywords:** Breast cancer, COVID-19, Resilience, Trait mindfulness, Self-efficacy, Optimism, Hope, Courage, Psychological distress, Anxiety

## Abstract

**Purpose:**

This study aims to understand the association between positive personal resources (i.e., optimism, hope, courage, trait mindfulness, and self-efficacy), resilience, and psychological distress (i.e., anxiety, depression, stress) in women with breast cancer and breast cancer survivors during the COVID-19 pandemic. We hypothesized that personal positive resources can directly influence resilience, which in turn prevented psychological distress.

**Methods:**

The research sample consisted of 409 Italian women (49% patients, 51% survivors) who were administered a questionnaire to assess positive resources, resiliency, and distress. structural equation model (SEM) analysis was carried out to confirm the hypothetical-theoretical model.

**Results:**

Personal positive resources had a direct positive effect on resilience, which prevented from distress. These results were observed across cancer patients and survivors, and regardless the level of direct exposure to COVID-19.

**Conclusions:**

In both patients and survivors, the relationships between positive personal resources, resilience, and psychological distress is strong enough to be not influenced by the level of exposure to COVID-19 and despite COVID-19 pandemic caused the disruption of active treatment plans and delays in routine check-ups.

**Implications for cancer survivors:**

Implications of this study suggest the urgency to screen positive resources and to identify women with lower resilience and a potentially higher susceptibility to develop psychological distress. For these women, our findings suggest the implementation of psychological interventions that build resilience.

## Introduction

Although the coronavirus disease 2019 (COVID-19) pandemic caused an unprecedented upheaval in the general population, it represented—and still represents—an even more worrisome time for vulnerable groups including both cancer patients and survivors [1, 2]. The immunosuppressive effects of cancer and its treatments [3], as well as the multimorbidity that often occurs in cancer patients and survivors [4, 5], have enhanced patients’ risk of contracting COVID-19 [6]. In addition, both cancer patients and survivors are at greater risks of experiencing more severe COVID-19 symptoms compared to the general population [2, 7, 8]. Alarmingly, cancer deaths have risen during the COVID-19 pandemic [9, 10].

Breast cancer represents both the most common type of cancer and cause of cancer death in women worldwide [11, 12]. Several studies showed that breast cancer is associated with psychological distress both in women recently diagnosed [13] and survivors [14, 15]. Common mental health concerns reported in both groups include depression, anxiety, and stress-related disorders [16]. The COVID-19 pandemic has exacerbated psychological distress among breast cancer patients and survivors [1, 17, 18]. These findings underline the urgency to identify protective psychological resources to mitigate the onset of these symptoms in the context of the COVID-19 pandemic. Resilience is considered a good candidate to reduce emotional distress [19] and to build upon through psycho-oncological interventions [19–21].

### Resilience and positive personal resources in breast cancer patients.

**Fig. 1:**
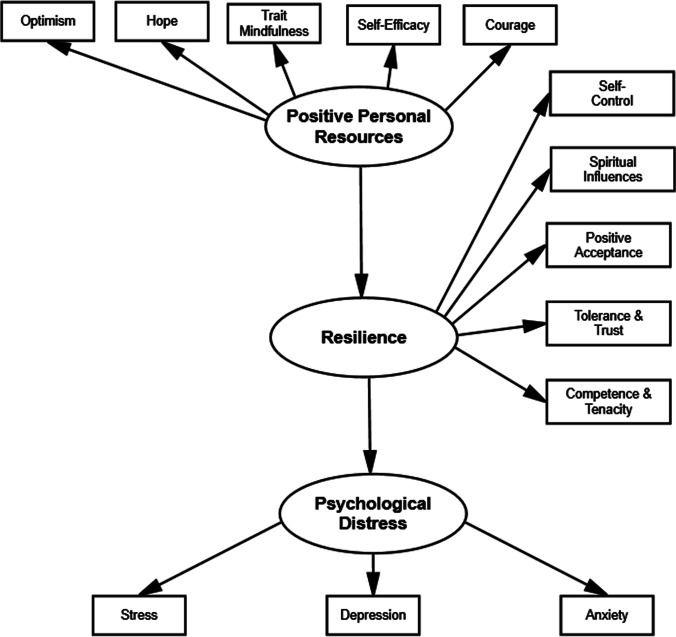
Model including positive personal resources to predict resilience and psychological distress in breast cancer women during the COVID-19 pandemic

Resilience is defined as the ability to maintain or restore relatively stable psychological and physical functioning when faced with stressful or adverse events [22, 23], such as having been diagnosed with cancer [24, 25] or coping with the COVID-19 pandemic itself [26]. A stressor that disturbs an individual’s homeostasis must be present in order to evaluate an individual’s personal resilience. Thus, resilience acts as a dynamic mechanism since it can change over time and can be influenced by several environmental factors [23, 27]. Interestingly, research demonstrated that resilience can be enhanced by positive personal resources—such as self-efficacy, optimism, hope, courage, and trait mindfulness—in breast cancer patients [19, 28–31]. Through the activation of affective, motivational, and behavioral mechanisms in difficult situations [32], such as receiving chemotherapy for breast cancer [31], self-efficacy (i.e., the belief of being able to perform new or difficult tasks and to achieve the desired results) can promote resilience [32]. Similarly, optimism (i.e., a stable predisposition to expect positive rather negative events will happen in one’s life), hope (i.e., to want that something to happen or be true), and courage (i.e., persistence or perseverance in the face of a dangerous situation despite feeling fears) increases resilience in breast cancer patients [19, 30, 31]. In addition, several studies demonstrated the effectiveness of mindfulness training in enhancing resilience in breast cancer women [28, 29]. Consequently, high levels of trait mindfulness (i.e., the predisposition to be mindful in day-to-day life) are expected to increase resilience [33]. As the influences of positive personal resources on resilience appears to be direct [19], it is possible to speculate that this relationship is not influenced by the time of cancer diagnosis and the level of exposure to the COVID-19 pandemic. In details:

- **Hp 1.** Positive personal resources positively predict resilience (see Fig. 1).
- **Hp 1a.** Positive personal resources positively predict resilience, regardless the time of the diagnosis and having experienced COVID-19 stressful events.

### Resilience and psychological distress in breast cancer patients

Several findings demonstrated that high levels of resilience act as a protective factor against the development of anxiety, stress, and depression in breast cancer patients and survivors [34, 36, 37]. However, the COVID-19 pandemic represents an alarming challenge for both groups as it resulted in the disruption of active treatment plans and delays in routine check-ups, respectively [1], thus resulting in enhanced psychological distress [1, 17, 18]. Interestingly, breast cancer survivors with high levels of resilience experienced less fear of COVID-19, despite their failure to maintain the medical follow-up due to the COVID-19 pandemic [38]. Thus, we hypothesize that resilience acts as a protective factor also against psychological distress during the second wave of COVID-19 regardless the time of breast cancer diagnosis (diagnosis received during the COVID-19 pandemic vs survivors) and the level of exposure to COVID-19 pandemic. Specifically:

- **Hp 2.** High levels of Resilience negatively predict psychological distress (see Fig. 1).
- **Hp 2a**. High levels of Resilience negatively predict psychological distress, regardless the time of the diagnosis and having experienced COVID-19 stressful events.

Despite the evidence discussed above, no studies to our knowledge have been performed to test the stability of positive personal resources and resilience and their key role in counteracting hardship and preventing distress in breast cancer patients and survivors in the context of the COVID-19 pandemic. Thus, the aims of this study were to investigate the (a) role of personal positive resources on resilience and (b) impact of resilience on psychological distress in breast cancer women during the second wave of COVID-19 pandemic. These relationships were investigated taking into account if patients were diagnosed with breast cancer before or after the start of COVID-19 (patients vs survivors) and if they had experienced COVID-19 stressful events (e.g., having contracted COVID-19 or/and having a family member infected by COVID-19).

## Materials and methods

### Participants

The sample was composed of 409 women living in different Italian regions (North 37.65%, Central 23.97%, South and Island 38.38%) with a mean age of 49 years, ranging from 25 to 76 years (SD = 10.01). Two hundred participants (49%) received a diagnosis of breast cancer during the COVID-19 pandemic whereas 208 (51%) were survivors. 156 (38.14%) had stage I, 173 (42.29%) had stage II, 62 (15.16%) had stage III, and 18 (4.41%) had stage IV disease. With respect to education, 201 (49.14%) participants held a high school diploma, 132 (32.27%) a university degree, 71 (17.36%) a middle school diploma, and 5 (1.22%) a primary school diploma. Regarding employment status, 258 (63.08%) participants were working, 48 were unemployed (11.73%), 6 (1.46%) were students, 38 (9.29%) retired, and 59 (14.42%) were homemakers. Regarding marital status, 116 (28.36%) were single and 293 (71.64%) were in a relationship.

### Measures

#### COVID-19 stressful events

Stressful vents related to COVID-19 were analyzed with five dichotomous questions presented in the biographical form of the protocol submitted (response alternatives: yes/no). The items were as follows: (1) “Have you contracted the flu during the COVID-19 pandemic?”; (2) “Have you been tested for COVID-19?”; (3) “Have you contracted COVID-19?”; (4) “Has anyone in your family contracted COVID-19?”; (5) “Have you been bereaved due to COVID-19?”. A total score was obtained summing answers (yes = 1) with higher score indicating more stressful events experienced.

#### Perceived Stress Scale (PSS-10)

Perceived Stress was evaluated through the Italian 10-item version of the Perceived Stress Scale (PSS-10) [39]. Each item, scored on a 5-point Likert scale ranging from 0 (never) to 4 (very often), investigates stressful experiences and responses to stress that occurred in the month before the detection (Cronbach’s α = 0.86). The global PSS-10 score ranges from 0 to 40, with higher scores indicating higher levels of perceived stress.

#### Hospital Anxiety and Depression Scale (HADS)

Anxiety and depression were analyzed with the Italian version of Hospital Anxiety and Depression Scale (HADS) [40]. Seven items assess depression (e.g., “I have lost interest in my appearance”), and another seven anxiety (e.g., “I feel tense or wound up”). The items were rated using a 4‐point Likert scale (from 0 to 3) with higher scores reflecting higher levels of depression and anxiety. Cronbach's α were 0.85 and 0.88 for the depression and anxiety scale, respectively.

#### Visions about future (VAF)

Optimism and hope were measured using the Vision About Future Scale [41]. It consists of 19 items and assesses attitudes toward hope, optimism, and pessimism. Participants responded to each item on a scale from 1 (it describes me not at all) to 5 (it describes me very well). The 19 items are divided into three subscales that measure optimism (6 items; e.g., “Usually, I am full of enthusiasm and optimism”), hope (7 items; e.g., “In the future I will do what I'm not able to do today”) and pessimism (6 items; e.g., “I will hardly find a job really suitable for me”). For the purposes of this study, only the scores of the optimism and hope subscales were considered. In our sample, Cronbach’s alpha for optimism and hope subscales were 0.90 and 0.92, respectively.

#### General Self-efficacy Scale (GSE)

Self-efficacy was assessed by using the Italian version of General Self-efficacy Scale (GSE) [42]. It consists in 10 items scored on a 4-point Likert scale ranging from 1 (not at all true) to 4 (exactly true) with higher score indicating higher perceived self-efficacy (Cronbach’s α = 0.91). Examples of items include “I can always manage to solve difficult problems if I try hard enough” and “I can solve most problems if I invest the necessary effort”.

#### Courage measure (CM)

Courage was evaluated by using the Italian version of the courage measure (CM) [43]. It consists in six items scored on a 7-point Likert-type scale ranging from 1 (never) to 7 (always). Higher scores reflect higher levels of perceived courage (Cronbach’s α = 0.87). Examples of items are “Even if I feel terrified, I will stay in that situation until I have done what I need to do” and “I try to get over my fears'”.

#### Mindful Attention Awareness Scale (MAAS)

Trait mindfulness were assessed thought the Italian version of Mindful Attention Awareness Scale (MAAS) [44]. Each of the 15 items, scored on a 6-point Likert scale ranging from 1 (almost always) to 6 (almost never), measure the extent to which individuals are attentive to the present moment in daily life (Cronbach’s α = 0.86). Examples of items are “I find myself doing things without paying attention” and “I find it difficult to stay focused on what’s happening in the present”. Higher scores indicate greater characteristics of mindfulness.

#### Connor-Davidson Resilience Scale (CD-RISC-25)

Resilience was measured with the Italian version of the Connor–Davidson Resilience Scale [45]. The 25 items were rated using a 5-point Likert scale ranging from 0 (not true at all) to 4 (true nearly all the time) and they are distributed in five sub-scales: positive acceptance (5 items), competence and tenacity (8 items), self-control (3 items), spiritual influences (2 items), and tolerance and trust (7 items). Examples of items are “able to adapt to change” (positive acceptance sub-scale), “can deal with whatever comes” (competence and tenacity sub-scale), “in control of your life” (self-control sub-scale), “sometimes fate or God can help” (spiritual influences sub-scale), and “you work to attain your goals” (tolerance and trust sub-scale). Higher scores indicate higher levels of resilience for total score and of each sub-scale. Internal consistency (Cronbach’s α) for the total scale, positive acceptance, competence and tenacity, self-control, and tolerance and trust were 0.94, 0.77, 0.90, 0.67, and 0.87, respectively.

### Procedure

Contact information for breast cancer survivors who were eligible to participate was obtained by psycho-oncologists (D.V.; M.V; M.C.; M.L.I.) operating in the voluntary association “Ali Rosa,” in Italy. A cross-sectional web-based survey design was adopted to cover the entire national territory, using the free software Google Forms®. The online survey was distributed during the second wave of COVID-19 pandemic between October 25th and December 28th of 2020. An information letter about the purpose of the study was mailed to all breast cancer patients and survivors together with a link including questionnaires on demographic-medical variables and study questionnaires. The participants were informed that participation in the study was voluntary, the survey was anonymous and confidential, and they could withdraw from the survey at any time. Additionally, an online consent form was completed by all participants. Approval for this study was obtained from the Ethical Committee of Calabria Region (Catanzaro, Italy).

### Data analysis

All analyses were conducted on SPSS and its extension Amos (version 27.0). Prior to conducting the analyses, we examined the missing values in the data. Listwise deletion was used when a case had more than 10% of missing answers [46]. Otherwise, the sample mean score was used to replace the missing value. Then, descriptive statistics and Pearson’s correlations were computed for the measured variables. Starting from the observed correlations, a structural equation model (SEM) was tested to estimate the magnitude and significance of the causal connections among a set of exogenous and endogenous variables. The model included 3 latent variables and 13 manifest variables (Fig. 1). The exogenous latent variable was named personal positive resources and it directly influenced resilience, the endogenous latent variable that was linked to psychological distress (the outcome latent variable). Positive personal resources were measured through optimism (VAF-O), hope (VAF-H), trait mindfulness (MAAS), self-efficacy (GSE), and courage (CM). Resilience was measured using the five scales of the CD-RISC-25 (self-control, spiritual influence, positive acceptance, tolerance-trust, and competence-tenacity). Depression, anxiety, and stress (as measured by the HADS and the PSS, respectively) were used as indicators of psychological distress. Goodness-of-fit was evaluated using χ2/df ratio, comparative fit index (CFI), Tucker-Lewis Index (TLI), and root mean square error of approximation (RMSEA). The relative chi-square should be less than 5 [47] and Byrne [48] recommended that a RMSEA approximately 0.08 and 0.06 and CFI and TLI above values of 0.90 and 0.95 would suggest moderate and excellent model fit, respectively. Multi-group SEM analysis was used to evaluate whether the model was consistent across the time of diagnosis and own COVID-19 experience. For the time of diagnosis, the sample was split in two groups: women who had received the diagnosis before (*N* = 279, 69.2%) vs after (*N* = 124, 30.8%) the start of the COVID-19 pandemic. For own COVID-19 experience, the COVID-19 stressful events score was dichotomized (scores ≥ 1 vs scores = 0) to obtain two groups: women who have (*N* = 245, 60.8%) and have not (*N* = 158, 39.2%) directly experienced COVID-19.

Invariance exists when at least two conditions are satisfied. First, the latent variables must be associated with the same set of observed variables in each group (measurement invariance). Second, the relationships between the latent variables must not be significantly different across groups (structural invariance). Thus, to assess measurement and structural invariance, a hierarchically nested series of SEM were applied. An unconstrained model was used as a baseline (baseline model) and five more restrictive models were tested. Specifically, measurement parameters were constrained to be equal across groups in model 1, and measurement and structural parameters were constrained to be equal across groups in model 2. Equality constrains were added for structural covariances and structural residuals in model 3 and model 4, respectively. Finally, measurement residuals were constrained to be equal in model 5. Models were compared using the chi-square difference statistic (Δ*χ*2) and the comparative fit index difference (ΔCFI) and root mean square error of approximation (ΔRMSEA) with values of ≤ 0.01 and ≤ 0.015, respectively, indicating no significant differences in nested models [49, 50]. Maximum likelihood estimation was utilized for all models.

## Results

### Preliminary analyses

Prior to conducting the analyses, we examined the missing values in the data. About 1.5% of the total cases (*n* = 6) were deleted listwise because more than 10% of the variable scores were missing. Three cases had 1 or 2 missing values. These values were replaced using the sample mean scores of the missing variables (specifically, we replaced two GSE scores and two CM scores). All the skewness and kurtosis indices ranged within − 1 and 1 (i.e., skewness ranged from − 0.79 to 0.72 and kurtosis from − 0.55 to 0.57) suggesting there was not a substantial departure from a normal distribution. These results indicate maximum likelihood estimation was appropriate for SEM. Finally, correlations supported the hypothesized pattern of relationships among the observed variables (Table 1).

**Table 1:** Pearson’s correlates between the variables in the study

	M	SD	(1)	(2)	(3)	(4)	(5)	(6)	(7)	(9)	(10)	(11)	(12)	(13)
(1)VAF-O	21.06	6.13	-											
(2)VAF-H	23.63	7.02	.75	-										
(3)MAAS	60.68	13.85	.28	.21	-									
(4)GSE	30.36	6.08	.57	.56	.25	-								
(5)CM	32.10	7.08	.42	.43	.28	.58	-							
(6)CDRS-1	23.43	6.60	.65	.64	.22	.67	.57	-						
(7) CDRS-2	16.39	5.32	.64	.64	.27	.67	.54	.82	-					
(9) CDRS-3	17.43	4.61	.62	.61	.26	.60	.45	.76	.76	-				
(10) CDRS-4	7.49	2.72	.54	.48	.27	.60	.47	.67	.70	.63	-			
(11) CDRS-5	5.84	2.03	.51	.52	.18	.49	.36	.72	.59	.82	.49	-		
(12)HADS-A	9.11	4.81	-.51	-.42	-.35	-.40	-.29	-.48	-.45	-.50	-.41	-.42	-	
(13) HADS-D	6.00	4.29	-.58	-.51	-.35	-.41	-.33	-.56	-.49	-.60	-.47	-.52	.74	-
(14)PSS	21.01	7.90	-.55	-.52	-.37	-.43	-.29	-.50	-.48	-.50	-.45	-.42	.70	.65

*Note*: *N* = 403. *HADS-D* = Hospital Anxiety and Depression Scale- Depression, *HADS-A* = Hospital Anxiety and Depression Scale-Anxiety, *PSS* = Perceived Stress Scale, *VAF-O* Vision About Future-Optimism, *VAF-H* Vision About Future-Hope, *CDRS-1* = Connor-Davidson Resilience Scale-Competence and Tenacity, *CDRS-2* = Connor-Davidson Resilience Scale-Trust and Tolerance, *CDRS-3* = Connor-Davidson Resilience Scale-Positive acceptance, *CDRS-4* = Connor-Davidson Resilience Scale–Self-control, *CDRS-5* = Connor-Davidson Resilience Scale-Spiritual Influence, *GSE* = General Self-Efficacy Scale, *MAAS* = Mindful Awareness Attention Scale, *CM* = Courage Measure. All correlations are significant at *p* < .001

### Single-group SEM analysis

The above-described model (Fig. 1) was tested. The initial model showed a poor fit to the data: *χ*2(63) = 478.2, *p* < 0.001, *χ*2/df = 7.59, CFI = 0.89, TLI = 0.87, RMSEA = 0.13. Modification indices suggested three additional covariance paths between these variables in the model: optimism and hope, spiritual influence and positive acceptance, and spiritual influence and tolerance-trust. Since optimism and hope are two sub-scales of the VAF scale, and the other ones are subscales of the CD-RISC-25, these results suggest the presence of a method factor (i.e., a systematic variance between the subscales of the same instrument that was not explained by the initial model). Thus, we added these covariance links and this modified model showed a good fit to the data: *χ*2(60) = 209.1, *p* < 0.001, *χ*2/df = 3.48, CFI = 0.96, TLI = 0.95, RMSEA = 0.08. Standardized measurement parameters were statistically significant and loaded onto its hypothesized latent variable (values ranged from 0.34 to 0.79 for positive personal resources, from 0.74 to 0.91 for resilience, and from 0.80 to 0.87 for psychological distress). The structural model shows a positive association between positive personal resources and resilience (*r* = 0.95, *p* < 0.001), and a negative relationship between resilience and psychological distress (*r* =  − 0.68, *p* < 0.001). Thus, as expected, personal positive resources, including optimism, hope, self-efficacy, trait mindfulness, general self-efficacy, and courage had a direct positive effect on resilience, which in turn prevented from psychological distress, i.e., anxiety, depression, and stress.

### Multi-group SEM analysis

The overall and comparative fit statistics of invariance models are presented in Table 2 and Table 3. When comparing the model across groups defined on the time of diagnosis (before vs. after the start of the COVID-19 pandemic), goodness of fit indices supported evidence for measurement and structural invariance, including measurement residual (Δ*χ*2 = 36.63, Δdf = 31, *p* = 0.224; ΔCFI = 0.001; ΔRMSEA = 0.004). When comparing the model across groups defined on own COVID-19 experience (i.e., having vs. having not directly experienced the effects of the infectious disease), goodness of fit indices supported evidence for measurement and structural invariance (Δ*χ*2 = 10.09, Δdf = 15, *p* = 0.814; ΔCFI = 0.002; ΔRMSEA = 0.004). When comparing cross-group equality of measurement residuals, the difference in *χ*2 values between models was significant at *p* < 0.05, but ΔCFI and ΔRMSEA were both 0.004, suggesting that there is also evidence for invariantce at the residual measurement level.

**Table 2:** Invariance fit statistics across groups defined on the time of diagnosis (before vs after the start of the COVID-19 pandemic)

Model	*χ2* (*df*)	*CFI*	*RMSEA*	Model comparison	Δχ2	Δ*df*	*p*	*ΔCFI*	*ΔRMSEA*
*Baseline*	279.91 (120)	.958	.058	*-*	*-*	*-*	*-*	*-*	*-*
*Model 1*	289.37 (130)	.959	.055	*Model 1 -Baseline*	9.47	10	.488	-.001	..003
*Model 2*	296.13 (132)	.957	.056	*Model 2 -Baseline*	16.23	12	.181	.001	.002
*Model 3*	298.47 (133)	.957	.056	*Model 3 -Baseline*	18.56	13	.137	.001	.002
*Model 4*	301.13 (135)	.957	.055	*Model 4 -Baseline*	21.22	15	.130	.001	.003
*Model 5*	316.54 (151)	.957	.052	*Model 5 -Baseline*	36.63	31	.224	.001	.004

*Note*: Before group: *N* = 279, After group: *N* = 124. *χ*^*2*^ = chi-square, *CFI* = comparative fit index, *RMSEA* = root mean square error of approximation, *Δχ*^*2*^ = difference in chi-squares between nested models, Δ*df* = difference in degrees of freedom between nested models, *p* = probability value of Δχ^2^ test, *ΔCFI* = difference between CFIs of nested models. *ΔRMSEA* = difference between RMSEAs of nested models. *Model 1* = equality of measurement weights, *Model 2* = Model 1 + equality of structural weights, *Model 3* = Model 2 + equality of structural covariances, *Model 4* = Model 3 + equality of structural residuals, *Model 5* = Model 4 + equality of measurement residuals

**Table 3:** Invariance fit statistics across groups defined on COVID-19 pandemic own experience (having vs having not directly experienced the effects of the infectious disease)

Model	χ2 (*df*)	*CFI*	*RMSEA*	Model comparison	Δχ0032	Δ*df*	*p*	*ΔCFI*	*ΔRMSEA*
*Baseline*	288.59 (120)	.956	.059	*-*	*-*	*-*	*-*	*-*	*-*
*Model 1*	294.40 (130)	.957	.056	*Model 1 -Baseline*	5.82	10	.830	.001	.003
*Model 2*	296.86 (132)	.957	.056	*Model 2 -Baseline*	8.28	12	*.*763	.001	.003
*Model 3*	297.41 (133)	.957	.056	*Model 3 -Baseline*	8.83	13	.786	.001	.003
*Model 4*	298.67(135)	.958	.055	*Model 4 -Baseline*	10.09	15	.814	.002	.004
*Model 5*	335.76 (151)	.952	.055	*Model 5 -Baseline*	47.17	31	.032	.004	.004

*Note*: Direct experience group: *N* = 245, No direct experience group: *N* = 158. *χ*^*2*^ = chi-square, *CFI* = comparative fit index, *RMSEA* = root mean square error of approximation, *Δχ*^*2*^ = difference in chi-squares between nested models, Δ*df* = difference in degrees of freedom between nested models, *p* = probability value of Δχ^2^ test, *ΔCFI* = difference between CFIs of nested models. *ΔRMSEA* = difference between RMSEAs of nested models. Model 1 = equality of measurement weights, *Model 2* = Model 1 + equality of structural weights, *Model 3* = Model 2 + equality of structural covariances, *Model 4* = Model 3 + equality of structural residuals, Model 5 = Model 4 + equality of measurement residuals

## Discussion

This study aims to better understand the association between positive personal resources (i.e., optimism, hope, courage, trait mindfulness, and self-efficacy), resilience, and psychological distress (i.e., anxiety, depression, stress) in women with breast cancer and survivors during the second wave of COVID-19 pandemic. Specifically, we hypothesized that personal positive resources can directly influence resilience which in turn prevented psychological distress. The analysis carried out confirmed the hypothetical-theoretical model. Aligned with previous studies performed outside of the COVID-19 pandemic [19, 28–31] positive personal resources increase the levels of resilience in breast cancer patients and survivors. Consistently with our results, literature also shows that resilience can reduce psychological distress in breast cancer patients and survivors [34–37]. Interestingly, our findings suggest that the relationship between positive personal resources, resilience, and psychological distress were not influenced by the level of exposure to COVID-19 (e.g., having contracted the COVID-19 or having suffered a bereavement due to COVID-19) and the time of breast cancer diagnosis (patients vs survivors), despite that the COVID-19 pandemic caused the disruption of active treatment plans and delays in routine check-ups in most cancer patients and survivors [1]. These results are in line with the original conceptualization of resilience of Rutter [51]: people with high resilience are affected by stressors similar to their low resilience counterparts, but they are able to maintain a certain emotional stability beyond personal negative experiences and unfavorable environmental conditions.

### The theoretical and practical contribution of the study

Considering that no studies have examined the impact of positive personal resources on resilience, as well as the effect of resilience on psychological distress in women with breast cancer and survivors during the COVID-19 pandemic, our results have enhanced the theoretical knowledge on this area of research. Specifically, the present study shows that personal positive resources can directly influence resilience and that higher levels of resilience prevent psychological distress despite the levels of exposition to COVID-19 and the time of diagnosis. Implications of this study suggest the urgency to screen resilience among breast cancer patients and survivors in order to early identify women with lower resilience and a potentially higher vulnerability to develop psychological distress. For these women, our findings suggest the implementation of psychological interventions that build resilience to effectively reduce psychological distress during the COVID-19 pandemic. For example, a pilot randomized clinical trial demonstrated the effectiveness of the Stress Management and Resilience Training (SMART) Program in building resilience among breast cancer patients [21].

### Limitations of the study

There are considerable limitations to this research that can be helpful for future studies. First, it is important to recognize that sampling used is not as effective as true random sampling; nonetheless, it allowed us to overcome specific disadvantages connected with true random sampling such as being overly expensive and time-consuming. Moreover, although path analysis was performed to examine “causal” hypotheses, cross-sectional data were collected and future studies would benefit from a longitudinal design. Lastly, self-reported measures were administered to assess the dimensions of this study. Future research should take into consideration different methods (e.g., clinician-ratings, peer-ratings) to reduce the influence of self-report bias.

## Conclusion

In sum, our results suggest that personal positive resources influenced resilience which in turn prevented from psychological distress, regardless the time of diagnosis and the own direct experience with the COVID-19 disease. In other words, we observed that during the second wave of COVID-19 pandemic resilience acted as a protective factor to cope with the stressful situation, and it was fostered by individual optimism, hope, self-efficacy, courage, and trait mindfulness. Additionally, these resources acted positively on resilience, which protected from anxiety, depression, and stress, despite having received the cancer diagnosis during the pandemic and having had close experiences with the COVID-19 disease.

## Data Availability

Data are available at https://osf.io/8jqf3/

## References

[1] Swainston J, Chapman B, Grunfeld EA, Derakshan N (2020) COVID-19 lockdown and its adverse impact on psychological health in breast cancer. Front psychol, 2033.10.3389/fpsyg.2020.02033 10.3389/fpsyg.2020.02033 PMC7476556 32982846

[2] Wang H, Zhang L (2020). Risk of COVID-19 for patients with cancer. Lancet Oncol.

[3] Verma R, Foster RE, Horgan K (2016). Lymphocyte depletion and repopulation after chemotherapy for primary breast cancer. Breast Cancer Res.

[4] Meneses K, Benz R, Azuero A, Jablonski-Jaudon R, McNees P (2015) Multimorbidity and breast cancer. In Seminars in oncology nursing 31(2):163–169. WB Saunders. 10.1016/j.soncn.2015.02.004 10.1016/j.soncn.2015.02.004 25951745

[5] Renzi C, Kaushal A, Emery J (2019). Comorbid chronic diseases and cancer diagnosis: disease-specific effects and underlying mechanisms. Nat Rev Clin Oncol.

[6] Al-Shamsi HO, Alhazzani W, Alhuraiji A (2020). A practical approach to the management of cancer patients during the novel coronavirus disease 2019 (COVID-19) pandemic: an international collaborative group. Oncologist.

[7] Carreir H, Strongman H, Peppa M (2020). Prevalence of COVID-19-related risk factors and risk of severe influenza outcomes in cancer survivors: a matched cohort study using linked English electronic health records data. EClinicalMedicine.

[8] Yang F, Shi S, Zhu J, Shi J, Dai K, Chen X (2020). Clinical characteristics and outcomes of cancer patients with COVID-19. J Med Virol.

[9] Lai AG, Pasea L, Banerjee A (2020). Estimated impact of the COVID-19 pandemic on cancer services and excess 1-year mortality in people with cancer and multimorbidity: near real-time data on cancer care, cancer deaths and a population-based cohort study. BMJ Open.

[10] Sud A, Jones ME, Broggio J (2020). Collateral damage: the impact on outcomes from cancer surgery of the COVID-19 pandemic. Ann Oncol.

[11] Azamjah N, Soltan-Zadeh Y, Zayeri F (2019). Global trend of breast cancer mortality rate: a 25-year study. Asian Pac J Cancer Prev: APJCP.

[12] Christensen H, Marck D (2017) The efficacy of mindfulness based stress reduction (MBSR) for decreasing anxiety and depression among breast cancer survivors. Sch Physician Assist Stud 16:613. https://commons.pacificu.edu/pa/613

[13] Schubart JR, Emerich M, Farnan M, Stanley Smith J, Kauffman GL, Kass RB (2014). Screening for psychological distress in surgical breast cancer patients. Ann Surg Oncol.

[14] Schreier AM, Johnson LA, Vohra NA, Muzaffar M, Kyle B (2019). Post-treatment symptoms of pain, anxiety, sleep disturbance, and fatigue in breast cancer survivors. Pain Manag Nurs.

[15] Zainal NZ, Nik-Jaafar NR, Baharudin A, Sabki ZA, Ng CG (2013). Prevalence of depression in breast cancer survivors: a systematic review of observational studies. Asian Pac J Cancer Prev.

[16] Yang H, Brand JS, Fang F (2017). Time-dependent risk of depression, anxiety, and stress-related disorders in patients with invasive and in situ breast cancer. Int J Cancer.

[17] Chen X, Wang L, Liu L (2021). Factors associated with psychological distress among patients with breast cancer during the COVID-19 pandemic: a cross-sectional study in Wuhan. China Support Care Cancer.

[18] Massicotte V, Ivers H, Savard J (2021). COVID-19 pandemic stressors and psychological symptoms in breast cancer patients. Curr Oncol.

[19] Ye ZJ, Peng CH, Zhang HW (2018). A biopsychosocial model of resilience for breast cancer: a preliminary study in mainland China. Eur J Oncol Nurs.

[20] Ye ZJ, Liang MZ, Qiu HZ (2016). Effect of a multidiscipline mentor-based program, Be Resilient to Breast Cancer (BRBC), on female breast cancer survivors in mainland China—A randomized, controlled, theoretically-derived intervention trial. Breast Cancer Res Treat.

[21] Loprinzi CE, Prasad K, Schroeder DR, Sood A (2011). Stress Management and Resilience Training (SMART) program to decrease stress and enhance resilience among breast cancer survivors: a pilot randomized clinical trial. Clin Breast Cancer.

[22] Bonanno GA, Westphal M, Mancini AD (2011). Resilience to loss and potential trauma. Annu Rev Clin Psychol.

[23] Seiler A, Jenewein J (2019). Resilience in cancer patients. Front Psych.

[24] Lin YH, Kao CC, Wu SF, Hung SL, Yang HY, Tung HY (2017). Risk factors of post-traumatic stress symptoms in patients with cancer. J Clin Nurs.

[25] Wu X, Wang J, Cofie R, Kaminga AC, Liu A (2016). Prevalence of posttraumatic stress disorder among breast cancer patients: a meta-analysis. Iran J Public Health.

[26] Silver RC (2020). Surviving the trauma of COVID-19. Science.

[27] Manne S, Ostroff J, Winkel G, Goldstein L, Fox K, Grana G (2004). Posttraumatic growth after breast cancer: patient, partner, and couple perspectives. Psychosom Med.

[28] Borgi M, Collacchi B, Ortona E, Cirulli F (2020). Stress and coping in women with breast cancer: unravelling the mechanisms to improve resilience. Neurosci Biobehav Rev.

[29] Heidarian A, Zahrakar K, Mohsenzade F (2016). The effectiveness of mindfulness training on reducing rumination and enhancing resilience in female patients with breast cancer: a randomized trial. Iran Q J Breast Dis.

[30] Vartak J (2015). The role of hope and social support on resilience in cancer patients. Indian J Mental Health.

[31] Yang JH, Kim OS (2016). The structural equation model on resilience of breast cancer patients receiving chemotherapy. J Korean Acad Nurs.

[32] Schwarzer R., Warner L.M. (2013) Perceived Self-Efficacy and its Relationship to Resilience. In: Prince-Embury S., Saklofske D. (eds) Resilience in Children, Adolescents, and Adults. The Springer Series on Human Exceptionality. Springer, New York, NY. 10.1007/978-1-4614-4939-3_10

[33] Kiken LG, Garland EL, Bluth K, Palsson OS, Gaylord SA (2015). From a state to a trait: Trajectories of state mindfulness in meditation during intervention predict changes in trait mindfulness. Personality Individ Differ.

[34] Dooley LN, Slavich GM, Moreno PI, Bower JE (2017). Strength through adversity: Moderate lifetime stress exposure is associated with psychological resilience in breast cancer survivors. Stress Health.

[35] Fradelos EC, Papathanasiou IV, Veneti A (2017). Psychological distress and resilience in women diagnosed with breast cancer in Greece. Asian Pac J Cancer Prev: APJCP.

[36] Markovitz SE, Schrooten W, Arntz A, Peters ML (2015). Resilience as a predictor for emotional response to the diagnosis and surgery in breast cancer patients. Psychooncology.

[37] Ristevska-Dimitrovska G, Filov I, Rajchanovska D (2015). Resilience and quality of life in breast cancer patients. Open Access Macedonian J Med Sci.

[38] Koral L, Cirak Y (2021). The relationships between fear of cancer recurrence, spiritual well-being and psychological resilience in non-metastatic breast cancer survivors during the COVID-19 outbreak. Psychooncology.

[39] Mondo M, Sechi C, Cabras C (2019). Psychometric evaluation of three versions of the Italian Perceived Stress Scale. Curr Psychol.

[40] Annunziata MA, Muzzatti B, Altoe G (2011). Defining hospital anxiety and depression scale (HADS) structure by confirmatory factor analysis: a contribution to validation for oncological settings. Ann Oncol.

[41] Ginevra MC, Sgaramella TM, Ferrari L, Nota L, Santilli S, Soresi S (2017). Visions about future: A new scale assessing optimism, pessimism, and hope in adolescents. Int J Educ Vocat Guidance.

[42] Sibilia L, Schwarzer R, Jerusalem M (1995) Italian adaptation of the general self-efficacy scale. *Resource document. Ralf Schwarzer web site. Accessed Oct*, *2*, 2020. 10.1037/t00393-000

[43] Ginevra MC, Santilli S, Camussi E, Magnano P, Capozza D, Nota L (2020). The Italian adaptation of courage measure. Int J Educ Vocat Guidance.

[44] Veneziani CA, Voci A (2015). The Italian adaptation of the Mindful Awareness Attention Scale and its relation with individual differences and quality of life indexes. Mindfulness.

[45] Di Fabio A, Palazzeschi L (2012). Connor-Davidson Resilience Scale: proprietà psicometriche della versione italiana [Connor-Davidson Resilience Scale: psychometric properties of the Italian version]. Counsel Giornale Italiano Ricerca Appl.

[46] Kline RB (2016). Principles and Practice of Structural Equation Modeling.

[47] Marsh HW, Hocevar D (1985). Application of confirmatory factor analysis to the study of self-concept: First- and higher order factor models and their invariance across groups. Psychol Bull.

[48] Byrne BM (2010) Testing for the factorial validity of a theoretical construct (First-Order CFA Model). In *Structural Equation Modeling with AMOS,* 2^nd^: 74–82. New York: Routledge.

[49] Chen FF (2007). Sensitivity of goodness of fit indexes to lack of measurement invariance. Struct Equ Modeling.

[50] Cheung GW, Rensvold RB (2002). Evaluating goodness-of-fit indexes for testing measurement invariance. Struct Equ Model.

[51] Rutter M (1993). Resilience: some conceptual considerations. J Adolesc Health.

